# Cost-effectiveness of physical activity interventions in cancer survivors of developed countries: a systematic review

**DOI:** 10.1007/s11764-021-01002-0

**Published:** 2021-02-23

**Authors:** Barbara E. Gubler-Gut, Johannes Pöhlmann, Aline Flatz, Matthias Schwenkglenks, Sabine Rohrmann

**Affiliations:** 1 Institute of Physiotherapy, Zurich University of Applied Sciences, Katharina-Sulzer-Platz 9, 8400 Winterthur, Switzerland; 2 Winterthur Institute of Health Economics, Zurich University of Applied Sciences, Gertrudstrasse15, 8401 Winterthur, Switzerland; 3 Swiss Cancer League, Effingerstrasse 40, 3001 Berne, Switzerland; 4 Institute of Pharmaceutical Medicine, University of Basel, Klingenbergstrasse 61, 4056 Basel, Switzerland; 5 Epidemiology, Biostatistics and Prevention Institute, University of Zurich, Hirschengraben 84, 8001 Zurich, Switzerland

**Keywords:** Cancer survivor, Physical activity, Cost-effectiveness, Cost-utility

## Abstract

**Purpose:**

Physical activity has been shown to improve survival and quality of life of cancer patients. Due to differences in patient populations, healthcare settings, and types of intervention, cost-effectiveness analyses of physical activity interventions in cancer survivors are difficult to compare. Available evidence from breast cancer survivor research has shown inconsistent results, and transfer of results to other types of cancer is not straightforward. This paper systematically reviewed current evidence on the cost-effectiveness of physical activity interventions in cancer survivors independent of cancer type compared to usual care or another experimental intervention.

**Methods:**

The literature search was conducted in seven databases and enhanced by a search for gray literature. Eligible studies were restricted to developed countries and assessed using the CHEERS, CHEC, and PHILIPS checklists. The study protocol was pre-published in PROSPERO.

**Results:**

Seven studies, five cost-utility, and two combined cost-utility/cost-effectiveness analyses fully met the inclusion criteria. They covered eight different types of cancer and various interventions. The cost-effectiveness analyses were of moderate to high methodological quality. A high probability of cost-effectiveness was reported in two analyses. One intervention appeared to be not cost-effective, and one to be cost-effective only from an organizational perspective. Three other analyses reported a cost-effectiveness better than US$ 101,195 (€ 80,000) per QALY gained.

**Conclusions:**

Physical activity interventions in cancer survivors of developed countries were cost-effective in some but not all clinical trials reviewed.

**Implications for Cancer Survivors:**

Cost-effectiveness of physical activity interventions appear to depend upon the intensity of the activity.

**Supplementary Information:**

The online version contains supplementary material available at 10.1007/s11764-021-01002-0.

## Introduction

Cancer is one of the leading causes of death worldwide. The incidence of cancer was 18.1 million cases in 2018 and is increasing [1]. In western healthcare systems, 5-year survival rates range from 5% for liver or lung cancers to 90% for breast cancer [2] and are still improving over time. Therefore, cancer often becomes a chronic condition, which remains a challenge for daily life.

Survivors have to contend with secondary effects, such as physical, psychological, or social impairments, either caused by the disease itself or by treatment [3]. An overall reduction in quality of life is common for several cancer types because of symptoms such as fatigue, pain, or functional disability [4–6], which in turn affect employment, family life, and recreation. The symptoms often persist for many years, leading to chronicity and multi-morbidity. Most survivors do not achieve previous levels of function and report prolonged fatigue, cognitive limitations, depression, anxiety, sleep problems, pain, or sexual dysfunction for up to ten years after diagnosis [7].

Lifestyle interventions directed at physical activity, diet, weight control, and cessation of smoking are thought to be effective in alleviating these detrimental effects and improving quality of life [8–11]. Physical activity interventions have a positive effect on the quality of life of cancer survivors [12] and are frequently promoted and well-established in rehabilitation programs to combat the secondary effects of cancer. It is well documented that physical activity reduces the risks of cancer recurrence, all-cause mortality, and secondary chronic diseases [13, 14].

Healthcare systems operate under constrained budget conditions and must consider the increasing demand for rehabilitation programs for cancer survivors critically. The goals of optimizing quality of life and preventing secondary chronic diseases must be combined with a focus on patient-specific care needs, within the constraints of the healthcare budget. Therefore, an understanding of financial spending and cost-effectiveness in cancer care is essential. Depending on cancer type, disease stage, and age, net costs to all payers in the USA were expected to range between US$ 20,000 and US$ 100,000 in the first year after diagnosis and to be lower in the extended survivorship phase, before increasing in end-of-life treatment [15]. Costs are driven by medical costs such as new cancer treatments and hospitalization and indirect costs such as absenteeism, job loss or disability pensions [15–17]. Out-of-pocket expenses for medical care could range from 7 to 11% of medical costs [18–21]. The individual financial strain affects the patient and their family substantially [22] and correlates to poor treatment adherence [23], worsening of symptoms [24], poor quality of life [25], and shorter survival [26].

Interventions, such as those directed at physical activity, must be evaluated carefully regarding their impact on private and/or healthcare system budget allocation and value for money. There is some evidence of cost-effectiveness in physical activity promotion in population-based programs [27]. In cancer rehabilitation, results remain unclear. A systematic review of cost-effectiveness studies of physical activity programs in a multidimensional setting including all types of cancer showed little available evidence [28]. In programs for breast cancer survivors, results remained unclear because of differences in patient populations and healthcare settings [29]. A tendency in the literature to focus on breast cancer limits the transferability of results to other cancer types [28]. The present study systematically reviewed the existing literature on the cost-effectiveness of physical activity interventions in cancer survivors independent of cancer type compared to usual care or another experimental intervention.

## Methods

The systematic review on the cost-effectiveness of physical activity interventions in cancer survivors focused on quality-adjusted life years (QALYs), costs, and incremental cost-effectiveness as outcome data. The predefined study protocol was registered in the international prospective register of systematic reviews (PROSPERO; CRD42019130284).

### Selection criteria

Publications included in this review had to be written in English and had to be clinical trial-based or decision-analytical model-based cost-effectiveness or cost-utility studies conducted in developed countries, as defined in the UN World Economic Situation and Prospects 2018 [30] (USA, Canada, Japan, Australia, New Zealand, and the states of Europe), to allow for sufficient comparability, limiting transferability to developing and/or emerging countries. The studies included were considered without time restrictions. Studies conducted in other countries or not meeting eligible study designs were excluded.

The eligible populations were cancer survivors over 18 years of age with histologically confirmed cancer diagnosis of any type, expected survival period of at least 1 year, and participating in either a physical activity intervention or referred to a comparator strategy which could either be usual care or another experimental intervention. Studies including patients not meeting the criteria of a cancer survivor [31] were excluded. Physical activity interventions not following the definition of Casperson [32] that focused predominantly on physiologic effects, such as cardiovascular and/or endurance and/or strength-training, were not considered in the study.

### Search strategy

PubMed/MEDLINE via Ovid, CINAHL, Cochrane Library, EMBASE, Centre for Review and Dissemination (CRD), EconLit, and Epistemonikos were searched electronically using predefined key words and medical subject headings (MeSH). Validated search strings of the InterTASC Information Specialists’ Sub-Group guidelines (ISSG) [33] were used for costs, focusing on optimization of sensitivity and specificity (95.0% and specificity of 95.6% [34] for MEDLINE and sensitivity of 98.4% and a specificity of 97.1% [35] for EMBASE). Advanced searches for gray literature were performed using website search functionality in Google Scholar and the BioRxiv preprint server. Finally, bibliography mining and cited reference searches using reference lists was undertaken [36]. Table 1 provides an overview of the search strategy used, which was adapted for the other sources.

**Table 1:** PubMed/MEDLINE search strategy

1	exp Entoplasmas/ or(neoplasm* or cancer* or tumor* or tumour* or carcinoma* or neoplasia* or leukemia* or melanoma* or sarcoma* or lymphoma* or malignan* or oncolog*).ti,ab.
2	exp Exercise/ or exp Exercise Therapy/ or exp Sports/exp or(exercis* or sport* or fitness or exertion* or endurance or gymnastic*).ti,ab. or(physical adj3 (activ* or training)).ti,ab.
3	exp Cost-Benefit Analysis/ or costs.ti,ab. or economic*.ti,ab.or (cost adj3 (effectiv* or efficien* or analy* or utility or benefi*)).ti,ab.
4	1 and 2 and 3
5	4 not(animals not humans).sh.

### Screening process

Citations of all search results were downloaded into a literature-management package (EndNote X7.8; Thomson and Reuters, Philadelphia, PA) and imported to the free web-based application Rayyan QCRI [37]. Title and abstract were screened by two authors independently. The same approach was used for full-text screening. Discrepancies were solved by discussion or third-party arbitration.

### Data extraction and quality assessment

The data extraction form was developed prior to conduct of the review, based on Centre for Reviews and Dissemination (CRD)-recommendations [38, 39], the Cochrane Handbook guidance [40], and ISPOR recommendations [41]. The form was tested prior to the review by two authors independently and was adjusted through discussion. Extracted study characteristics included author, year of publication, country of study performance, study design, population, type of intervention, and comparator. To cover economic evaluation, data on study perspective, analytical approach, time horizon, direct and indirect costs, effectiveness, and cost-effectiveness were added. Data extraction was performed by one reviewer and verified by a second.

### Quality assessments

Quality of reporting and quality of methodology were determined using different checklists for cost-effectiveness analyses and were assessed by one reviewer and verified by a second. The Consolidated Health Economic Evaluation Reporting Scale (CHEERS) [42] was used to gather information on the quality of reporting. For the quality assessment of primary clinical trial-based cost-effectiveness analyses, the extended Consensus Health Economics Criteria (CHEC) checklist [43] was used, while for decision-analytic model-based analyses the guidelines for good practice in decision-analytic modeling (Philips) [44] were used. Both checklists (CHEC [43] and Philips [44]) were employed to assess studies using a combined trial-based and decision-analytic model-based approach. As recommended by the GRADE guidelines [45] and to ensure the confidence in effect estimates, the Cochrane Rob2 tool [46] was used to assess the risk of bias in the underlying clinical trials, where appropriate.

### Synthesis

Results were summarized in tables and graphically represented in cost-effectiveness planes. Subgroups of studies were formed based on type of intervention, starting point of intervention during medical treatment process, intensity and type of cancer. Specifically, interventions were subdivided into the categories of direct (face-to-face), indirect, or combined support groups. Intensity was subdivided into low intensity (up to 12 Borg [47]/65% of one repetition max. [48, 49]), moderate intensity (13–15 Borg [47]/66–79% of one repetition max. [48, 49]) or high intensity (16–20 Borg [47]/80–100% of one repetition max. [48, 49]) groups. Intervention starting point was either during or post medical treatment (radiotherapy and/or chemotherapy).

Cost data extracted from the studies were inflated to 2017 US Dollars using purchasing power parity (PPP) conversion factors [50]. In studies not reporting price year data, it was assumed that the price year was 1 year prior to study publication. Reporting was undertaken following PRISMA guidance [51].

## Results

**Fig. 1:**
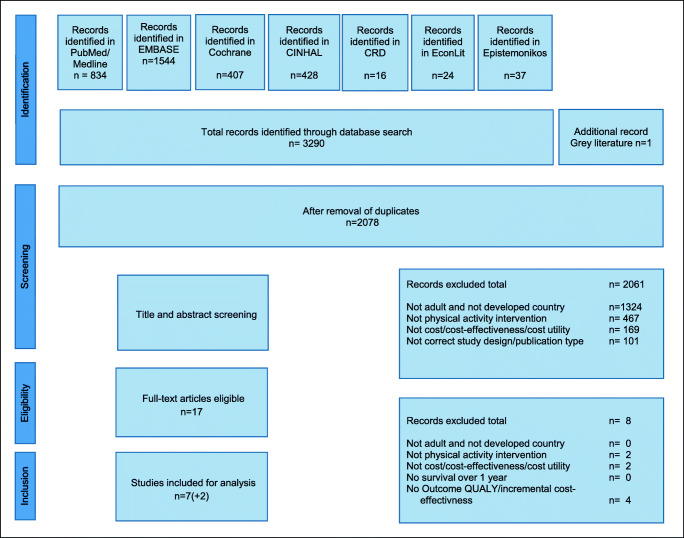
Study selection

The literature search was conducted in May 2019. A total of 3290 articles were identified. The gray literature search yielded one additional record, which has been published in the meantime [52]. Deduplication resulted in 2.078 remaining articles, of which 2061 were excluded in the title and abstract screening. The full text of the remaining 17 articles was analyzed, resulting in the exclusion of a further eight articles. No more articles were identified from bibliography mining and cited reference search (Fig. 1). Seven publications were finally included in the review: May et al. [53], Gordon et al. [54], van Waart et al. [55], Kampshoff et al. [56], Mewes et al. [57], Haines et al. [58], and Ha et al. [52] met all inclusion criteria. Broderick et al. [59] reported an incomplete cost-effectiveness analysis due to the unavailability of survival information, and Gordon et al. [60] presented a cost-consequences analysis. Both papers were included in the discussion.

### Study characteristics and participants

Five cost-utility analyses [52, 53, 56–58] and two combined cost-utility/cost-effectiveness analyses [54, 55] were published between 2010 and 2019 in the Netherlands, Australia, USA, and Ireland. Study characteristics are presented in Table 2 (see extended online Table 2 for more detail). A trial-based approach was chosen by five research groups [53–56, 58]. Mewes et al. [57] combined a trial-based and model-based approach, and Ha et al. [52] carried out a model-based analysis. The various clinical studies underlain the cost-effectiveness evaluations included a total of 3494 patients, ranging from 89 [58] to 1635 [52] participants per study. Four studies reported a breast cancer [54, 55, 57, 58], one study a lung cancer [52] and two studies a mixed cancer population [53, 56]. The mean age of the study populations was between 48.2 [57] and 78.9 [52] years and covered a wide range of baseline physical fitness level.

**Table 2:** Summary of study characteristics

	May et al. [53]	Gordon et al. [54]	van Waart et al. [55]	Kampshoff et al. [56]	Mewes et al. [57]	Haines et al. [58]	Ha et al. [52]
Year of publication	2017	2017	2018	2018	2015	2010	2019
Country	Netherlands	Australia	Netherlands	Netherlands	Netherlands	Australia	USA
Study design	CT-based CUA	CT-based CUA and CEA	CT-based CUA and CEA	CT-based CUA	AM and CT-based CUA	CT-based CUA	AM-based CUA
Population	165 (f) breast cancer; 18 (m) and 11 (f) colon cancer	194 (f) breast cancer	230 (f) breast cancer	181 (f) breast; 49 colon; 12 ovarian; 26 lymphoma; 4 cervix and 5 testis cancer (55 (m) and 41 (f))	422 (f) breast cancer	89 (f) breast cancer	547 (f) and 551 (m) lung cancer
Sample characteristics	Mean age 50.0 years ± 7.9 int.; 49.4 years ± 7.6 contr. in breast cancer; mean age 57.4 years ± 11.2 int. and 59.1 years ± 8.9 contr. in colon cancer	Mean age 52 ± 8 years	Mean age 51 years	Mean age HI, 53 years, and LMI, 55 years	Mean age 48.2 years	Mean age 55.9 years int.; 54.2 years contr.	Mean age 78.9 years
Setting	Outpatient clinic (7 center)	Home based, telephone based (4 center)	In-hospital based, home based (12 center)	NI	CBT, hospital based; PE, home based	Home based	Study center based, home based (8 center)
Intervention specification	D 60 min; I 45–65% of one repetition max; F 2 (supervised); DP 18 weeks	Fit for Future: D 45 min; I NI; F 4; DP: 32 weeks; telephone session: D 45 min ; I NI; F 4 (telephone support 16 times); DP 48 weeks	Onco-Move: D 30 min; I low; F 5; DP mean 17 weeks; OnTrack: D 45min; I moderate to high 80% of one rep. max.; F 2; DP mean 17 weeks	HI: D 60 min; I high; F 2; DPm 12 weeks; LMI: D 60 min; I low to moderate; F 2; DP 12 weeks	CBT: D 90 min every 6 weeks; I NA; D 12 weeks; PE: D 150 to 180 min; I 60–80% of one rep. max; F NI; DP 12 weeks	D 15–45 min; I 60–80% of VO_2_max; F NI; DP 18 weeks	D 60min; I Borg 13 for walking and 15/16 for exercise; F 2–4; DP 125 weeks
Intervention type	Cardiovascular interval and strength training, 30 min physical activity recommendation on 3 days a week	cardiovascular and strength training	Cardiovascular and strength training, physical activity recommendation of 30 min being active 5 times a week	Cardiovascular and strength training, recommendation of 30 min being physically active on 3 days a week	Cardiovascular training	Cardiovascular and strength training, shoulder training	Cardiovascular and strength training with flexibility and balance components
Adherence to the intervention	83%	88%	Onco Move: NI class intervention, 55% home-based training; OnTrack: 71% class intervention, 48% home-based training	HI, 74%; LMI, 70%	CBT, 58%; PE, 64%; CBT/PE,70%	Higher in the first 3 months than later on. After 12 months, 11 of 37 participants completed their program	PA 63%
Starting point of intervention	< 6 weeks for breast and < 10 weeks for colon cancer after diagnosis	3-4 weeks post-surgery	First cycle of chemotherapy until 3 weeks after the last cycle	Completed adjuvant chemotherapy	undergone adjuvant chemotherapy and/or hormonal therapy	Following surgery undergoing adjuvant chemo therapy	Possible walk of 400 m within 15 min without assistive device or sitting
Comparator	Usual care	Usual care	Usual care	Waiting list control	Waiting list control	Active sham intervention	Weekly health education
Comparator specification	NI	NI	NI	NI	NI	D 30min I; NA F NI	NI

*CT* clinical trial, *AM* analytic model, *CUA* cost-utility analysis, *CEA* cost-effectiveness analysis, *f* female, *m* male, *int.* intervention, *contr.* control, *F* frequency per week, *I* intensity, *D* duration, *HI* high intensity, *DP* duration of the program, *LMI* low to moderate intensity, *CBT* cognitive behavioral therapy, *PE* physical exercise, *NI* no information, *NA* not applicable

### Intervention characteristics

Interventions were launched either during the cancer treatment or after completion of the primary therapy, ranging from as early as 6 to 10 weeks after cancer diagnosis [53] to a start after completed chemotherapy [56, 57]. Intervention intensity and duration ranged from low to moderate [55] to high intensity [56] and from twelve [56, 57] to 125 weeks [52]. In the control groups, cancer survivors received either usual care [54, 55], usual care with instructions to maintain habitual levels of activity [53], waiting list control [56, 57], or active sham intervention with relaxation program or weekly health education [52, 58]. Interventions focused on cardiovascular [57] or combined cardiovascular and strength [52–56, 58] training respecting the needs of the patient.

Activities in single sessions varied in duration and frequency. Participants were physically active between 30 [55] to 60 min [53, 58] per session, from two [54, 57], to five [55] times a week, in a range from low to moderate [58] to high [55–58] intensity. Adherence to the training varied between 48 [55] and 88% [54].

### Quality assessments

#### Quality assessment of cost-effectiveness studies

Quality of reporting was moderate to high [52–58], and was affected by missing or incomplete cost data [54, 55], insufficient information about discount rates [54, 55, 58], and unclear adjustment of unit cost estimates to a base year [54, 56, 58] (Table 3).

**Table 3:** Quality of reporting and methodology

	May et al. [53]	Gordon et al. [54]	van Waart et al. [55]	Kampshoff et al. [56]	Mewes et al. [57]	Haines et al. [58]	Ha et al. [52]
Quality of reporting CHEERS	High	Moderate	High	High	High	Moderate	High
Quality of methodology CHEC	High	Moderate	High	High	High	Moderate	NA
Quality of methodology Philips	NA	NA	NA	NA	High	NA	High

*NA* not applicable

Moderate to high quality of methodology was found overall for the studies by May et al. [53], Gordon et al. [54], van Waart et al. [55], Kampshoff et al. [56], Mewes et al. [57], and Haines et al. [58] (Table 3). Weaknesses identified were that some important and relevant costs for alternatives were not identified [54, 57], that not all data were reported, and that not all costs were valued appropriately [58]. Ha et al.’s [52] model-based cost-effectiveness analysis and the Mewes et al. [57] paper, as a combination of trial-based and model-based analysis, were considered to be of high methodological quality.

#### Quality assessment of RCTs underlying the cost-effectiveness studies

**Fig. 2:**
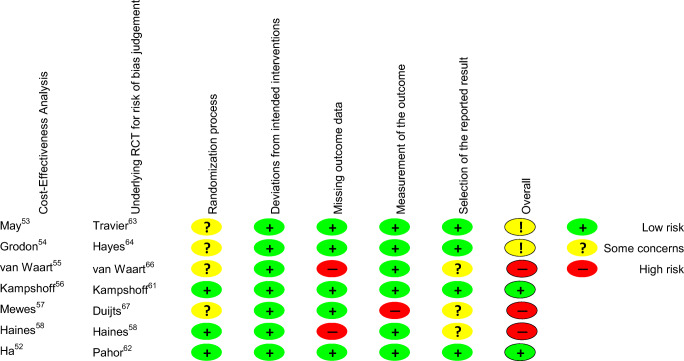
Risk of bias judgment

The RCTs of Kampshoff et al. [61] and Pahor et al. [62] were considered to have low risk of bias due to implementation of a sham intervention (Fig. 2). Some concerns in the studies by Travier et al. [63] and Hayes et al. [64] were lack of assessor blinding. High risk of bias in the studies by van Waart et al. [65], Duijts et al. [66], and Haines et al. [58] was based on lack of information on concealment of the allocation sequence and high losses to follow-up.

### Cost-effectiveness

**Fig. 3:**
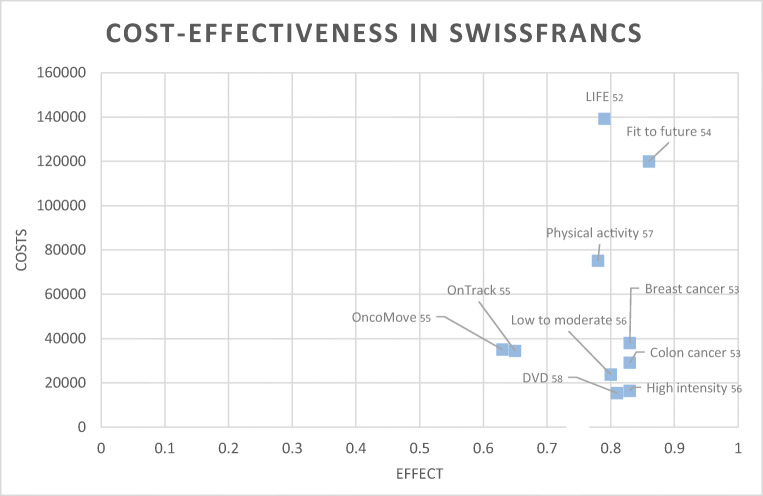
Cost-effectiveness in Swiss Francs

Cost-effectiveness results are summarized in Table 4 (see extended online Table 4 for more detail) and on the cost-effectiveness plane in Fig. 3. High-intensity training in the study of Kampshoff et al. [56], physical activity interventions in colon [53] and lung [52] cancer patients, were reported to be cost-effective. For breast cancer, inconsistent results were shown. Mewes et al. [57] showed cost-effectiveness up to a ceiling ratio of US$ 36,229/QALY gained. Gordon et al. [54], van Waart et al. [55], and Haines et al. [58] and their colleagues demonstrated a likelihood of being cost-effective under 45%. Personal-supported programs delivered by physiotherapists or exercise physiologists [52, 53, 55, 56] were more likely to be cost-effective than self-management programs [54, 57, 58].

**Table 4:** Summary of study-level cost-effectiveness data

	May et al. [53]	Gordon et al. [54]	van Waart et al. [55]	Kampshoff et al. [56]	Mewes et al. [57]	Haines et al. [58]	Ha et al. [52]
Analytic approach	CT based	CT based	CT based	CT based	CT and DAM based	CT based	DAM based
Perspective	sp, hcp	hpp, spp, pp	sp	sp	hsp	sp	op, sp
Definition of treatment effect	QALY	QALY, improvement in Quality of life	QALY, improvement in clinical outcome	QALY	QALY	QALY	QALY, disease-free survival
Primary health outcome cost-effectiveness analysis (utility score)	EQ-5D after 18 weeks for breast cancer: int. 0.83 utilities; contr. 0.83 utilities; EQ-5D after 18 weeks for colon cancer: int. 0.83 utilities; contr. 0.80 utilities	EQ-5D-3L 12-month post-surgery int. 0.86 utilities; contr. 0.85 utilities	EQ-5D-3L after 6 month OncoMove 0.63 utilities; OnTrack 0.65 utilities	EQ-5D-3L global quality of life after 64 weeks LMI 0.8 utilities; HI 0.83 utilities	SF 36 converted to EQ-5D 0.78 utilities from the first to 0.85 utilities to the last cycle with transition probabilities in CBT of 0.484 utilities and 0.453 utilities in PE	EQ-5D utility after 6 month int. 0.80 -utilities; contr. 0.83 utilities	EQ-5D generates an average of 0.79 utilities
Currency	US Dollars	US Dollars	US Dollars	US Dollars	US Dollars	US Dollars	US Dollars
Cost year	2017	2017	2017	2017	2017	2017	2017
Time horizon	Less than 1 year	1 year	1 year	64 weeks	5 years	Less than 1 year	Median of 2.6 years
Discounting	No discounting	No discounting	No discounting	Costs and effects	Costs and effects	No discounting	Costs and effects
Discount rate	NA	NA	NA	Future costs at 4%, effects at 1.5% annually	Future costs at 4%, effects at 1.5% annually	NR	3%
Health care costs intervention group	sp mean of 29142 in breast cancer, 22296 in colon cancer; hcp mean of 18880 in breast cancer and 11346 in colon cancer *without intervention costs	spp 98244; pp 87108 *with intervention costs	OncoMove mean 29335; OnTrack mean 28884 *without intervention costs	LMI 19375; HI 13356 *without intervention costs	CBT 697/patient, for all 86 patients 59971; PE 707 per patient, 61506 for all patients after 1 cycle *with intervention costs	Mean 10668 *without Intervention costs	op 110224; sp 116685 *NI if intervention costs were included or not
Intervention costs	PACT in breast cancer mean 1179; PACT in colon cancer mean 1224	spp 751; pp 650	Onco-Move 4493; OnTrack 73906	LMI 1189; HI: 1252	CBT 22777 all patients; PE 23879 all patients	NA	NA
Health care cost control group	sp mean of 25949 in breast cancer, 28446 in colon cancer; hcp mean of 24259 in breast cancer, 27436 in colon cancer	2079	26304	NR	NR	Mean 4041	op 105485; sp 105967
Indirect costs intervention group	sp mean of 1640 unpaid domestic help, 6501 sick leaves in breast cancer; mean of 1761 unpaid domestic help, 7258 sick leaves in colon cancer	NR	OncoMove 23888; OnTrack 22510	LMI 34415; HI 27389	NR	0	op 6461
Indirect costs control group	sp mean of 1387 unpaid domestic help, 5655 sick leaves in breast cancer; mean of 610 unpaid domestic help, 8652 sick leaves in colon cancer	NR	23170	NR	NR	Mean 1355	op 482
Threshold value in 2017 US Dollar	101195	34615	101195	25299/65777	NA	NA	100000
Incremental cost per strategy	4325 breast cancer; 6417 colon cancer	spp 2051; pp 1771	OncoMove NR; OnTrack NR	3544 HI versus LMI	CBT 256; PE 258	0	4740
Incremental effectiveness	0.01 in breast cancer; 0.03 in colon cancer	0.009 in service provider model	OncoMove 0.04; OnTrack 0.04	No within group differences	CBT 0.0079; PE 0.0067	0.03	0.06
Sensitivity analysis	Probabilistic, deterministic	Probabilistic, deterministic	Probabilistic, deterministic	Probabilistic, deterministic	Probabilistic, deterministic	Probabilistic, deterministic	Probabilistic, deterministic
Main cost-effectiveness results	599.083/QALY breast cancer; NI colon cancer	spp 81648/QALY; pp 70483/QALY	OncoMove 88611/QALY; OnTrack 34047/QALY	128.163/QALY	CBT 25.969/QALY; PE 39.124/QALY	NI	79504/QALY
Summary of results described by the authors of primary papers	Colon cancer: lower health care costs and less hours absence from work; Breast cancer: higher cost, no apparent effect on quality of life	Exercise intervention may be cost-effective if society is willing to pay approximately 34615 US Dollar per month	OnTrack could be cost-effective for general and physical fatigue depending on willingness to pay; OncoMove is not likely to be cost-effective	Effect on role and social functioning is larger for HI than LMI; Cardiorespiratory fitness was successfully for LMI and HI; HI was cost-effective due to lower health care costs	CBT and PE are effective and cost-effective	DVD multimodal exercise program improve short term health but of questionable economic efficiency	Costs of exercise program were most sensitive to the change of results and the intervention; cost-effective on an organizational but not on a societal level
Probability of cost-effectiveness	Colon cancer intervention was 100% dominant; breast cancer with a probability of cost-effectiveness of 2%	Likelihood of spp being cost-effectiveness was 44.4% and of pp 46.3%	Max. probability of Onco-Move and OnTrack being cost-effectiveness at 6 month follow-up was 17% and 31%, respectively; With 101,195 willingness-to-pay threshold 55% and 79%, respectively	Probability of HI exercise being cost-effective compared with LMI was 87%; probability increases to 91% at a willingness to pay of 29184/QALY	PE has the highest probability of being cost-effective up to a ceiling ratio of 32888/QALY; beyond CBT has the highest probability of being cost-effective with a probability of 49% at 42802/QALY up to 56% at 111472/QALY	Low probability of both less costly and more effective than the control condition	The LIFE was cost-effective with a 71% (with willingness to pay threshold of 150,000/QALY, 94%) probability and usual care with a probability of 27%

*CT* clinical trial, *DAM* decision-analytic model, *int*. intervention group, *contr*. control group, *sp* societal perspective, *hcp* health care perspective, *hpp* health provider perspective, *spp* service provider perspective, *pp* private perspective, *hsp* health system perspective, *op* organizational perspective, *NR* not reported, *NA* not applicable, *HI* high intensity, *LMI* low to moderate intensity, *CBT* cognitive behavioral therapy, *PE* physical exercise, *SD* standard deviation, *QALY* quality adjusted life years, *SA* sensitivity analysis

### Breast cancer

An Australian study reported a moderate- to high-intensity 32-week physical activity intervention with an ICER of US$ 81,648/QALY from the service provider perspective and US$ 70,483/QALY from the private perspective, respectively. With a threshold of US$ 34,615/QALY, the probability of cost-effectiveness was 44.4% and 46.3% [54] in the probabilistic sensitivity analysis. Sensitivity analysis indicated that ICERs were sensitive to EQ-5D-3L weights when varied within their 95% confidence intervals [54]. Another study from the Netherlands conducted 17-week physical activity programs of both low intensity and of moderate- to high-intensity and observed an ICER of US$ 88,611/QALY and the moderate to high-intensity training an ICER of US$ 34,047/QALY. With a threshold of US$ 101,195/QALY, the probability of cost-effectiveness ranged from 55% for low intensity to 79% for moderate to high-intensity training [55]. In a sensitivity analysis including solely compliant participants, probability of cost-effectiveness seemed to be better [55]. The study by Mewes et al. [57], a 12-week individualized physical activity training, showed an ICER of US$ 39,124/QALY for the physical exercise strategy. The exercise intervention had the highest probability of being cost-effective up to a willingness-to-pay of US$ 32,888/QALY. Beyond this value, the second intervention assessed (cognitive behavioral therapy) had a higher probability of being cost-effective. Results were robust, showing lower cost-effectiveness with shorter treatment duration [57] in a sensitivity analysis. Haines et al. undertook a moderate to high-intensity 18-week DVD-delivered physical activity intervention without reporting any QALY values transparently [58].

### Breast and colon cancer

A Dutch 18-week low to moderate physical activity intervention for breast and colon cancer showed an ICER of US$ 599.083/QALY. In breast cancer, the probability of being cost-effective was 2 to 6% with a willingness-to-pay threshold of US$ 101,195/QALY [53]. Sensitivity analyses with different willingness to pay thresholds showed higher costs for a small additional effect on QALY. Therefore, the physical activity intervention was shown to be cost-effective for colon, but not breast cancer patients [53].

### Lung cancer

A US study assessing a moderate to high-intensity training over 125 weeks in lung cancer survivors showed an incremental cost-effectiveness ratio of US$ 79,504/QALY and led to a probability of being cost-effective of 71% with a willingness to pay threshold of US$ 100,000/QALY and of 94% with US$ 150,000/QALY [52]. With costs of US$ 116,686, the moderate- to high-intensity training [52] reported the highest program expenditures seen in this review, for a long-term intervention. In this study, the model was most sensitive to the costs of the exercise program, probability of increasing exercise, and health utility benefit related to exercise [52]. The intervention was cost-effective from an organizational perspective, including costs for targeted therapy but not immunotherapy. No cost-effectiveness was seen from a societal perspective, including participant opportunity costs related to time spent in exercise [52].

### Mixed cancer population

A Dutch trial, comparing high- and low-to-moderate-intensity training of 12 weeks, indicated a significant effect of high-intensity physical activity on role and social functioning. Cardiorespiratory fitness showed a short- and long-term increase in the low-to-moderate- and high-intensity groups [56]. An ICER of US$ 128.163/QALY was indicated for the high-intensity-training compared to the low to moderate-intensity training [56]. With a willingness-to-pay of US$ 25,299/QALY, the probability of cost-effectiveness was 91% and 95% with a willingness-to-pay of US$ 65,777/QALY [56]. Sensitivity analyses for payment of all scheduled exercise sessions and disease recurrence were robust [56]. High-intensity training was cost-effective and reduced healthcare costs [56].

## Discussion

This systematic review assessed the cost-effectiveness of physical activity interventions in cancer survivors, including any type of cancer. Seven cost-utility and/or cost-effectiveness studies [52–58] were included and systematically analyzed. These studies evaluated eleven different types of interventions, in four settings and at three different levels of intensity. Studies were of moderate to high methodological and reporting quality and the risk of bias of underlying clinical trials ranged from low to high. Whereas results for breast cancer were unclear [53–55, 57, 58], physical activity interventions for lung cancer patients were reported to be cost-effective, with 71% probability at a willingness-to-pay threshold of US$100,000/QALY rising to 94% at a US$150,000/QALY willingness-to-pay [52]. For colon cancer, results were reported to be dominant [53] and for a high-intensity training, a probability of cost-effectiveness of 91% at a willingness-to-pay threshold of US$ 25,299/QALY was reported [56]. Studies indicating cost-effectiveness of physical activity interventions were of high quality of reporting and methodology [52, 53, 55, 56] with low to high risk of bias [58, 61, 62, 65, 66].

Our results are in line with previous reviews. Mewes et al. reported ICER’s below the prevailing willingness-to-pay threshold in multidimensional rehabilitation programs applied to various types of cancer [28]. In 2012, they found four economic evaluations published between 2005 and 2011 with significant benefits of the intervention over the control group in terms of QALYs, energy, fear of regression, mood, and pain. However, comparability between the studies found was low due to different types of interventions. Khan et al. focused their review on breast cancer survivors and documented contrasting conclusions due to heterogeneity in the interventions delivered [29]. Guillon and colleagues discussed unclear results of three cost-effectiveness analyses of physiotherapy-led exercise programs for breast and head and neck cancer patients [67, 68].

The identified findings for colon cancer [53, 56] are underlined by documented effects of physical activity interventions on quality of life in colon and colorectal cancer survivors [59, 60, 69, 70], but further reliable data on cost-effectiveness are missing. The same applies to lung cancer. With a probability of being cost-effective of 94% with a willingness-to-pay threshold of US$150,000 per QALY [52], the intervention studied by Ha and colleagues in lung cancer was cost-effective [52]. This is in line with the effectivity of physical activity interventions in lung cancer but with no further information on cost-effectiveness [71].

Van Dongen et al. included patients with hematologic malignancy treated with stem cell transplantation and trained them in a supervised 18-week high-intensity interval and resistance training [72]. They found the intervention to be not cost-effective due to lack of clinical effectiveness [72]. One reason could have been suboptimal compliance or the timing of the intervention, due to the length of time to recovery in stem cell transplantation [72], which is a problem in comparability between different types of cancer.

Effects of physical activity interventions are driven by intensity, duration/adherence, delivery mode, and starting point of intervention during rehabilitation. This has implications for cost-effectiveness. We found high-intensity interventions, such as described by van Waart et al. [55] and Kampshoff et al. [56], may be more cost-effective relative to usual care than light to moderate physical activity programs, due to a potential reduction in healthcare use [56]. This is in line with studies on effectiveness which compared a low-volume and high-intensity to low-to-moderate-intensity training or usual care. They included different types of cancer and described an effect on quality of life (*d* = 1.11; 95% CI 0.50, 1.72), cardiorespiratory fitness (*d* = 0.97; 95% CI 0.36, 1.56), lower body strength (*P* < 0.01; *d* = − 0.83; 95% CI − 1.40, − 0.22) and waist circumference (*P* = 0.01; *d* = − 0.48; 95% CI − 1.10, 0.10) [73]. The result is underlined by another study, which delivered a well-tolerated, high-intensity intervention over 20 weeks in lung cancer patients, showing significant effects on peak oxygen uptake (3.4 mL/kg/min between-group difference, 95% CI 3.3 to 6.7; *p* < 0.001), total muscle strength (leg press increased by 27.4 ± 26.2 kg (*p* = 0.001)), functional fitness and quality of life (after intervention, the QoL scale was 51.8 ± 5.5 in the exercise group and 43.3 ± 11.3 in the control group (*p* = 0.006)) compared to usual care [74].

Two thirds of cancer survivors do not meet physical activity recommendations in the USA [75], in particular women of low education and with comorbid conditions [76]. Program duration and adherence to intervention could play important roles in the cost-effectiveness of physical activity interventions such that long-term support might result in better adherence to training and less hospitalizations [75]. However, in our review, intervention duration, varying from 12 to 125 weeks, did not correlate with cost-effectiveness. Adherence to physical activity interventions, which ranged from 48% for home-based activities [55] to 83% for supervised classes [53], was also not correlated with cost-effectiveness.

Delivery modes of physical activity programs vary widely, from personal support to distance-based interventions. In our analysis, the interventions that were cost-effective [52, 53, 55, 56] were personal support programs delivered by a home-based additional training or recommendation. However, the starting point of the physical activity intervention did not seem to affect cost-effectiveness. Patients diagnosed with cancer often report difficulties in the adoption and maintenance of exercise. Concerns about safety, desire for professional guidance, physical limitations, fatigue, or lack of time were reported [77–79]. This indicates that personal support could be beneficial. A systematic review of 27 distance-based physical activity interventions in cancer survivors found no effect on reported physical activity [80]. Goode et al. reported an effect of non-face-to-face lifestyle interventions in three quarters of the 27 studies, with a preference for telephone-based activities [81]. Novel technologies, with the possibility of delivering physical activity interventions to meet patients’ needs with an optimal allocation of resources, should be investigated. Furthermore, cost-effectiveness of program duration, frequency and intensity needs to be observed in-depth to allocate resources in an optimal manner. Last but not least, information about maintenance of long-term adherence is needed.

The strength of this study is that it uses a robust methodological procedure, based on clear eligibility criteria and standardized, validated assessment instruments. Two independent reviewers and a professional librarian were involved in the definition of the search process and analysis undertaken. Thorough analyses of different aspects of interventions were performed and the implications for cost-effectiveness assessed. There are some limitations to point out. Due to the heterogeneity of the identified studies researchers were unable to summarize the results quantitatively. The population in the trials underlying most of the cost-effectiveness analyses were not representative of all patients with the respective cancer types, with those included in the trials probably at an advanced stage of cancer, at an older age, more likely to be female and more active [82–84]. Due to inclusion of studies only from developed countries, transferability to developing and/or emerging countries is not possible. Further physical activity arrangements outside the study protocols were not assessed or reported. With respect to the reporting of cost-effectiveness, relevant information was not necessarily available from all studies, particularly for direct costs, indirect costs, and productivity losses. The economic burden of early retirement, productivity loss, and disability pensions is substantial [85].

## Conclusion

We systematically reviewed cost-effectiveness analyses of physical activity interventions in cancer survivors over all types of cancer. High-intensity training interventions appeared to have a potential for being cost-effective and two studies found that interventions for colon, respectively, lung cancer were cost-effective. Further results are inconclusive because of the heterogeneity of interventions and cost data available. More research is needed to make results more robust. A greater focus on cost-effectiveness studies considering different intervention characteristics, such as, frequency, intensity, duration, and intervention delivery modalities, could deliver more in-depth results. Furthermore, future work will need to cover software-assisted tools and wearables.

## Supplementary Information

ESM 1 (XLSX 19 kb)

ESM 2 (XLSX 21 kb)

## Data Availability

Systematic review—all data is publically available
